# Satisfaction of patients with diabetic kidney disease with traditional chinese medicine physician visits

**DOI:** 10.1016/j.heliyon.2022.e12371

**Published:** 2022-12-16

**Authors:** Wanling Zeng, Hong Chang Tan, Huang Fang Zheng, Amanda Rui Lin Lam, Kok Keong Teo, Chieh Suai Tan, Jean-Paul Kovalik, Sujoy Ghosh, Xiao Hui Xin

**Affiliations:** a Department of Endocrinology, Singapore General Hospital, Singapore; b Singapore Thong Chai Medical Institution, Singapore; c SingHealth Medicine ACP, Singapore General Hospital, Singapore; d Department of Renal Medicine, Singapore General Hospital, Singapore; e Cardiovascular and Metabolic Disease Program, Duke-NUS Medical School, Singapore; f Centre for Computational Biology, Duke-NUS Medical School, Singapore

**Keywords:** Patient satisfaction, Diabetic kidney disease, Traditional Chinese medicine

## Abstract

**Background:**

Patient-centred care is an important part of quality healthcare and patient satisfaction has been shown to be associated with improved clinical outcomes. We aim to explore the satisfaction of patients with diabetic kidney disease (DKD) with their visits to the TCM physician and its association with patients' socio-economic characteristics.

**Methods:**

A questionnaire survey was conducted among patients aged >21 years with DKD. Participants' demographic, socioeconomic characteristics and satisfaction scores measured with the self-administered Medical Interview Satisfaction Scale (MISS) were collected after they visited the TCM physician. MISS is a 26-item questionnaire consisting of three domains – cognitive, affective and behavioural which was developed to assess patient satisfaction with medical consultation. Independent samples t-test and one-way analysis of variance (ANOVA) were used to analyse the data.

**Results:**

137 participants completed the questionnaires and were included in the analysis. The mean satisfaction score was 3.1 out of 5, with the cognitive domain being significantly higher compared to the affective and behavioural domains. The mean satisfaction score of the cognitive domain differed significantly among participants staying in different types of housing and those with previous TCM encounters. The mean satisfaction score of the behavioural domain differed significantly among participants of different ethnicities. The mean satisfaction scores of all the domains were also significantly different among participants with different duration of follow-up with their TCM physicians.

**Conclusion:**

We found that ethnicity, types of housing, previous TCM experience and the duration of follow-up with the TCM physician may affect the satisfaction scores of patients with DKD.

## Highlights

• Patient perspectives and satisfaction have been shown to be associated with improved clinical outcomes.
• Patient satisfaction is multidimensional and consists of domains related to cognitive, affective and behaviour.
• Communication, addressing patients' concerns and encouraging greater patient participation in healthcare encounters are important.
• Ethnicity, type of housing, previous TCM experiences and the duration of follow up affect patient satisfaction.

## 1 Introduction

Diabetic kidney disease (DKD) is a major microvascular complication of diabetes and has become a global health issue with its increasing prevalence. The prevalence of DKD in patients in the local outpatient setting has been reported to be 53% [1]. DKD is associated with increased cardiovascular morbidities and mortalities and is the leading cause of end-stage renal failure (ESRF) [2]. The increase in healthcare utilisation has resulted in rising economic burden.

Management of DKD is challenging and a holistic approach is required for patient care. Conventional medicine is disease-targeted and has been proposed to be less interpersonal compared to complementary and alternative medicine (CAM) which concentrates on the harmony and restoration of balance between the body and mind [3, 4]. Patient-centred care focuses on the delivery of holistic care that individualises to patients' needs, values and experiences and is an increasingly important part of quality healthcare [5, 6]. Patient perspectives and satisfaction have been shown to be associated with improved clinical outcomes [7, 8]. Patients with higher satisfaction scores are more likely to adhere to their medical treatment and scheduled medical appointments. Patient satisfaction builds trust in the patient-healthcare professional relationship and allows for the continuation of care when the patient remains in the same healthcare organisation.

The measurement of patient satisfaction has attracted growing attention and interest in recent decades. With the increasing emphasis on patient-centred care, more healthcare regulators are incorporating patient satisfaction as a key component of quality assessment [9, 10, 11]. The determination of patient satisfaction and perspectives allows for quality improvement which ultimately affects the organisation and delivery of healthcare and patient outcomes [12]. The measurement of patient satisfaction is fraught with challenges and complexity. There are various validated qualitative and quantitative tools derived to measure patient satisfaction that were widely used such as the Medical Interview Satisfaction Scale (MISS) [13], Consumer Assessment Health Plans (CAHPS) [14] and the Patient Satisfaction Questionnaires (PSQ) [15].

Traditional Chinese medicine (TCM) is one of the oldest forms of CAM that is commonly practised worldwide and is highly regarded in Asian societies such as Taiwan, Hong Kong and Singapore. Even though both Western medicine and TCM are widely available in Singapore, there is a lack of integration and corroboration between TCM and the standard healthcare system. Some studies showed that patients choose to visit TCM as they are not satisfied with the care and treatment outcomes provided by allopathic medicine [16]. However, other studies demonstrated that patients use TCM because of cultural and personal healthcare beliefs rather than being dissatisfied with conventional medicine [3, 17]. Nonetheless, there are limited studies that evaluate patient satisfaction with TCM visits. Patient satisfaction is multidimensional and consists of domains related to cognitive, affective and behaviour. TCM consultation can have different impacts on one or more of the domains. Of interest, Singapore is a multicultural society with individuals belonging to different socio-economic and cultural backgrounds which can affect their satisfaction with TCM consultation. As such, we aim to assess the satisfaction with TCM consultation among patients with DKD as well as to examine the influence of socio-economic factors on the satisfaction scores.

## 2 Methods

### 2.1 Study population

This prospective study was conducted at Singapore General Hospital (SGH) and Singapore Thong Chai Medical Institute from 18 May 2015 to 7 Dec 2016. Electronic medical records of the participants were reviewed and those that satisfied the inclusion and exclusion criteria were invited to participate in the study. Eligible participants were patients above 21 years of age, with type II diabetes mellitus (T2DM) and chronic kidney disease (CKD) stage II to V. Patients with dementia, recent increase in serum creatinine of more than two-fold, non-diabetic kidney disease, renal replacement therapy, chronic liver diseases, or severe cardiac failure were excluded. Eligible participants were scheduled for their TCM consultation at Singapore Thong Chai Medical Institute. The purpose of the TCM visit was to diagnose and assess the severity of DKD and no treatment was given [18]. The study and research protocol was approved by the SingHealth Centralized Institutional Review Board (CRIB) (CIRB Reference: 2015/2004) and written informed consents were obtained from all study participants.

### 2.2 Patient satisfaction score

At the end of the TCM visit, each participant was given a copy of the MISS-26 questionnaire for self-administration at home. Questionnaires were either in English or Chinese language and participants were required to mail the questionnaires back to the hospital’s investigator team using a pre-prepared stamped envelope. MISS-26 was developed in the United States by Wolf et al. [19] in 1978 to investigate patient satisfaction with their medical consultation. MISS-26 is the first version of the questionnaire that has three subscales (cognitive, affective and behavioural). Participants have to choose among the 5 possible options on the Likert type scale: 5 – strongly agree, 4 – agree, 3 – uncertain, 2 – disagree, and 1 – strongly disagree. MISS-26 is chosen for its internal consistency, clear structure and central focus on the quality of doctor-patient interaction.

Demographic data related to the participants' age, gender, race, housing types, education, employment status and prior TCM experience were also collected with the questionnaires. 80% of the resident population in Singapore live in public housing called Housing and Development Board (HDB) flats and the rest in private housing. Among the various housing types, 5-room HDB flats and private housing are considered the most expensive. Thus, the housing types provide a surrogate indicator of the socioeconomic status of the participants.

### 2.3 Analysis

MISS-26 comprises of a total of 26 questions for the 3 domains with 9 questions for the cognitive domain, 9 questions for the affective domain and 8 questions for the behavioural domain. The mean overall score was calculated by dividing the total score by 26 items. The average satisfaction scores under each domain (cognitive, affective and behavioural) were calculated by dividing the total scores by the number of questions under each domain. The domain-specific scores were compared using one-way analysis of variance (ANOVA) and the post-hoc correction was done with the Bonferroni test. Independent samples t-test and ANOVA with post-hoc analysis were used to assess the effect of categorical variables such as gender, marital status, employment status, educational level, types of housing, languages spoken, prior history of TCM usage and duration of follow up with the TCM physicians on the mean MISS-26 satisfaction scores.

Missing data consisted of less than 10% of the overall responses and were omitted in the statistical model. The level of statistical significance was set at p < 0.05 and SPSS version 21 was used for all analyses.

## 3 Results

### 3.1 Demographic and socioeconomic characteristics

Of the 200 participants enrolled, 137 participants completed and returned their questionnaires and were included in the analysis. Demographic and socioeconomic characteristics and history of TCM usage are described in Table 1. The average age of the participants was 64 and 61.3% of the participants were male. The majority of the participants were Chinese (80.3%) and about half of the participants (54%) had previous encounters with TCM physician. Among those who had ever seen a TCM physician, 83.8% of the participants had been on follow up with their TCM physicians for less than a year.

**Table 1:** Patients' demographic and socioeconomic characteristics, N = 137.

		Frequency (percentage)
Mean age, years (SD)	64.1(8.3)	
Gender	Female	53 (38.7%)
	Male	84 (61.3%)
Race	Chinese	110 (80.3%)
	Malay/Indian/Others	27 (19.7%)
Education	Primary or lower	40 (29.2%)
	Secondary or lower	50 (36.5%)
	Post-secondary (non- tertiary)/tertiary	47 (34.3%)
Type of housing	1-/2-/3- room HDB	32 (23.4%)
	4-Room HDB	49 (35.8%)
	5-Room HDB/Private	55 (40.1%)
Employment Status	Working full-time	48 (35%)
	Working part-time	20 (14.6%)
	Not working (e.g., students, retirees, housewives & unemployed)	68 (49.6%)
Spoken Language	English	53 (38.7%)
	Mother Tongue	60 (43.8%)
	English and Mother Tongue	24 (17.5%)
Ever seen a TCM physician	Yes	74 (54%)
Duration of follow up with TCM physician	Less than a year	62 (83.8%)
	More than a year	12 (16.2%)

### 3.2 Satisfaction score.

The mean overall satisfaction score was 3.1 out of 5. The mean satisfaction scores of the cognitive, affective and behaviour domains were significantly different (p < 0.05) with the satisfaction score of the cognitive domain being the highest followed by the affective domain and the behavioural domain having the lowest satisfaction score (Table 2).

**Table 2:** Mean satisfaction score of the 3 domains and post-hoc analysis (Bonferroni).

Domain	Mean (out of 5 points) (SD)	Post-hoc analysis		
			Mean Difference (95% CI)	Sig.
Cognitive	3.4 (0.9)	Cognitive vs Affective	0.240 (0.036–0.443)	**0.015**
Affective	3.1 (0.6)	Affective vs Behaviour	0.477 (0.274–0.681)	**0.000**
Behaviour	2.6 (0.5)	Behaviour vs Cognitive	-0.717 (-0.514 – -0.920)	**0.000**

∗The mean difference is significant at the 0.05 level.

### 3.3 Comparison of satisfaction scores with different demographic and socioeconomic characteristics

The mean satisfaction score of the cognitive domain was significantly higher in participants staying in 4-room HDB compared to 5-room HDB and private housing (3.65 vs 3.11, p = 0.005) (Table 3) and higher in those with previous TCM consultation (3.53 vs 3.21, p = 0.038) (Table 4). Participants of Chinese ethnicity had a lower satisfaction score under the behavioural domain compared to their non-Chinese counterparts (2.60 vs 2.82, p = 0.048) (Table 4). Participants who had seen and been on follow up with their TCM physician for less than a year had a lower average score across all three domains compared to participants who had been on follow up for more than a year (2.89 vs 3.47, p = 0.001) (Table 4). Demographic and socioeconomic characteristics such as gender, age, marital status, employment status, education level and language spoken did not affect the MISS-26 satisfaction scores significantly (Tables S1–S4). We conducted a sensitivity analysis to evaluate the effect of excluding the missing data by conducting an imputation of the missing data using average values of the different domains and found no difference in the results.

**Table 3:** Difference in cognitive satisfaction score vs types of housing with post-hoc analysis (Bonferroni).

Housing types	Mean cognitive satisfaction score (SD)	Post-hoc analysis		
		Housing type	Mean Difference (95% CI)	Sig.
1-3 room HDB	3.31 (0.9)	1-3 room HDB vs 4-room HDB	-0.335 (-0.811–0.142)	0.273
4-room HDB	3.65 (0.7)	4-room HDB vs 5-room HDB/pte	0.541 (0.130–0.950)	**0.005**
5-room HDB/private	3.11 (0.9)	1-3 room HDB vs 5-room HDB/pte	-0.206 (-0.673–0.260)	0.854

∗The mean difference is significant at the 0.05 level.

**Table 4:** Difference in satisfaction score between those with and without prior TCM experience, Chinese vs. Malay/Indian/Others race and those who followed up with the TCM physician for >1 year vs < 1 year.

Satisfaction score	Previous TCM experience			Ethnicity			Duration of follow up with the TCM physician		
	No N = 63	Yes N = 74	P	Chinese N = 110	Malay/Indian/others N = 27	P	<1 year N = 62	>1 year N = 12	P
	Mean (SD)	Mean (SD)		Mean (SD)	Mean (SD)		Mean (SD)	Mean (SD)	
Cognitive	3.53 (0.90)	3.21 (0.87)	**0.038**	3.42(0.80)	3.11 (1.20)	0.102	3.07 (0.84)	3.95 (0.62)	**0.001**
Affective	3.18 (0.66)	3.07 (0.60)	0.350	3.12 (0.59)	3.14 (0.78)	0.841	3.00 (0.59)	3.45(0.49)	**0.017**
Behavioural	2.66 (0.52)	2.63 (0.53)	0.662	2.60(0.50)	2.82 (0.57)	**0.048**	2.57 (0.54)	2.93 (0.27)	**0.028**
Overall	3.14 (0.63)	2.98 (0.56)	0.119	3.06 (0.55)	3.03 (0.77)	0.799	2.89 (0.54)	3.47 (0.36)	**0.001**

Data presented as mean (SD) p <0.05.

## 4 Discussion

This is one of the few studies that investigate in patients with DKD, their satisfaction with TCM consultation. TCM has been highly regarded and widely practised worldwide including Singapore. Socio-economic background especially in a multicultural society such as Singapore can have a profound influence on patient satisfaction. Good patient-doctor interaction and active involvement of patients in self-management have shown to improve satisfaction, better adherence to medical care and ultimately better health outcomes [20, 21]. In our study, 54% of the participants had prior TCM consultation which was higher than the prevalence of 22% reported previously [17]. We found that the mean satisfaction score was the highest for cognitive domain followed by affective and behavioural domain. Participants with prior TCM experience and those who are staying in larger public flats or private housing had lower satisfaction in the cognitive domain, Chinese participants had lower satisfaction in the behavioural domain and participants with shorter duration of follow up with their TCM physician had lower satisfaction in all three domains.

The cognitive domain focuses on communication and explanation of the medical conditions, diagnosis, prognosis and treatment options by the physician to ensure understanding of the information by the patients [19]. It correlates significantly with the communication and exchanges of information at the end of the consultation. As TCM consultation emphasises the mutual exchange of verbal information and interpersonal doctor-patient relationship, patients were more satisfied with the cognitive and affective components of the encounter. Communication between healthcare providers and patients has long been established to be a critical component doctor-patient relationship and healthcare delivery, especially in chronic disease management [22]. Communication breakdown can adversely affect patient satisfaction and treatment outcomes [23]. The affective domain refers to the patient’s perception of the treatment relationship which includes trust and confidence in the healthcare provider and the willingness of the healthcare provider to listen to the patient’s concerns [19]. It correlates significantly with the initial interview process and focuses on the humanness of the encounter. By contrast, the behavioural domain evaluates the healthcare provider’s professionalism, physical examination, investigations, treatments and provision of medical advice which focuses on the diagnostic process [19]. The higher satisfaction score for the affective domain compared to the behavioural domain in our study might be related to the nature of the TCM physician visit, which was to establish a diagnosis of DKD using TCM methods and not for the provision of treatment.

Interestingly, in our study, those with previous TCM encounters have a lower mean satisfaction score in the cognitive domain than those without previous TCM encounters. The practice of TCM physicians in Singapore is regulated and TCM practitioners are expected to have high-level professionalism that is similar to western-medicine trained physicians [24]. Patients' expectations and satisfaction are influenced by their previous experience and those with prior TCM encounters might have expectations which were not met in the consultation that was solely diagnostic in nature [25]. We found that participants who owned a 5-room HDB or private housing had a lower mean satisfaction score in the cognitive domain compared to those who owned a 4-room HDB. Home ownership can be regarded as a proxy indicator of the financial status of the participants and a lower socioeconomic status has been associated with lower patients' satisfaction in prior study [26] which is incongruent with our findings.

We also found that Chinese participants had a lower mean satisfaction score in the behavioural domain compared to their non-Chinese counterparts. This finding is unexpected as an earlier study found that patients with race concordance with their healthcare providers generally reported a higher level of satisfaction and trust [27]. The influence of socioeconomic status and ethnicity will need to be examined in future studies.

Our study demonstrated that patients who have seen and been on follow up with their TCM physicians for T2DM for a longer duration had higher satisfaction scores in all three domains. This is supported by a previous study that found that longer patient-doctor relationship was associated with higher patient satisfaction and confidence due to continuity of care [28]. In our study, we found no difference in the satisfaction scores with regard to gender, age, marital status, employment status, education level and language spoken. Some studies demonstrated that older patients have a higher satisfaction score compared to younger patients [16, 29] while others found that females were more satisfied with their TCM consultation [4].

### 4.1 Strengths and limitations

Our study represents the first report describing in patients with DKD, their satisfaction with TCM visits in a multi-ethnic society where TCM and conventional medicine co-exist. Our findings highlight the importance of communication, addressing patients' concerns and encouraging greater patient participation in healthcare encounters to improve self-care management and patient satisfaction [22].

The study has several limitations. Firstly, the TCM visit is solely for diagnostic purposes as such it does not reflect the range of services and treatments provided by the TCM physicians which can affect the satisfaction score of the patients. Secondly, the study only includes patients with DM and CKD as such the results cannot be generalized to patients with other medical conditions or acute conditions. Thirdly, although MISS has good internal consistency and is widely used to evaluate patient satisfaction, it has not been validated in the local population.

## 5 Conclusion

In conclusion, we found that ethnicity, types of housing, previous TCM experience and the duration of follow up with the TCM physician affect the satisfaction scores of patients with DKD. Future studies comparing the satisfaction score between conventional care and TCM would allow us to gain a deeper insight into patients' perception of the 2 different systems. With increasing emphasis on patient-centred care and individualised medicine, patients satisfaction will be an important component in the assessment of the quality of healthcare.

## Declarations

### Author contribution statement

Hong Chang Tan: Conceived and designed the experiments; Performed the experiments; Contributed reagents, materials, analysis tools or data; Analyzed and interpreted the data.

Huang Fang Zheng, Amanda Rui Lin Lam, Kok Keong Teo, Chieh Suai Tan, Jean-Paul Kovalik: Performed the experiments; Contributed reagents, materials, analysis tools or data.

Sujoy Ghosh, Xiao Hui Xin: Analyzed and interpreted the data.

Wanling Zeng: Analyzed and interpreted the data; Wrote the paper.

### Funding statement

Hong Chang Tan was supported by 10.13039/501100001350Ministry of Health -Singapore [TCMCRG/3103004].

### Data availability statement

Data will be made available on request.

### Declaration of interest’s statement

The authors declare no competing interests.

### Additional information

No additional information is available for this paper.

## Appendix A Supplementary data

Supplementary tables Supplementary tables

DN Care Survey English version 1_ 2015 DN Care Survey English version 1_ 2015

MISS questionaires MISS questionaires
