# Supplementary material for: Satisfaction of patients with diabetic kidney disease with traditional chinese medicine physician visits
Source: Heliyon. 2022 Dec 16;8(12):e12371. doi: 10.1016/j.heliyon.2022.e12371 (PMC9800549; doi:10.1016/j.heliyon.2022.e12371)
Supplement: Supplementary tables [file mmc1.docx]

| Table S1: Satisfaction score vs gender | | | |
| --- | --- | --- | --- |
| Satisfaction score | Gender | | P |
|  | Male (N = 84) | Female (N = 53) |  |
|  | Mean (SD) | Mean (SD) |  |
| Cognitive | 3.44 (0.84) | 3.22 (0.97) | 0.148 |
| Affective | 3.12 (0.62) | 3.12 (0.65) | 0.991 |
| Behavioural | 2.67 (0.54) | 2.60 (0.50) | 0.430 |
| Mean | 3.10 (0.56) | 2.99 (0.64) | 0.321 |

| Table S2: Satisfaction score vs martial status | | | | |
| --- | --- | --- | --- | --- |
| Satisfaction score | Marital Status | | | P |
|  | Single (N = 15) | Married (N = 105) | Divorced/Separated/widowed (N = 16) |  |
|  | Mean (SD) | Mean (SD) | Mean (SD) |  |
| Cognitive | 2.99 (1.16) | 3.41 (0.87) | 3.43 (074) | 0.241 |
| Affective | 2.95 (0.83) | 3.14 (0.61) | 3.17 (0.47) | 0.514 |
| Behavioural | 2.59 (0.64) | 2.64 (0.53) | 2.74 (0.28) | 0.705 |
| Mean | 2.85 (0.82) | 3.08 (0.58) | 3.13 (0.44) | 0.341 |

| Table S3: Satisfaction score vs education level | | | | |
| --- | --- | --- | --- | --- |
| Satisfaction score | Education Level | | | P |
|  | Primary or lower (N = 40) | Secondary or lower (N = 50) | Post-Secondary (N = 47) |  |
|  | Mean (SD) | Mean (SD) | Mean (SD) |  |
| Cognitive | 3.42 (0.88) | 3.51 (0.86) | 3.15 (0.93) | 0.119 |
| Affective | 3.17 (0.63) | 3.18 (0.63) | 3.02 (0.66) | 0.409 |
| Behavioural | 2.71 (0.48) | 2.66 (0.45) | 2.56 (0.62) | 0.389 |
| Mean | 3.11 (0.58) | 3.13 (0.58) | 2.92 (0.61) | 0.167 |

| Table S4: Satisfaction score vs employment status | | | | |
| --- | --- | --- | --- | --- |
| Satisfaction score | Employment Status | | | P |
|  | Working full-time (N = 48) | Working part-time  (N = 20) | Not working  (N = 68) |  |
|  | Mean (SD) | Mean (SD) | Mean (SD) |  |
| Cognitive | 3.27 (0.87) | 3.73 (0.84) | 3.29 (0.90) | 0.112 |
| Affective | 3.11 (0.66) | 3.37 (0.57) | 3.04 (0.60) | 0.123 |
| Behavioural | 2.65 (0.59) | 2.78 (0.48) | 2.59 (0.48) | 0.340 |
| Mean | 3.02 (0.59) | 3.31 (0.57) | 2.99 (0.58) | 0.087 |
