# Supplementary material for: Satisfaction of patients with diabetic kidney disease with traditional chinese medicine physician visits
Source: Heliyon. 2022 Dec 16;8(12):e12371. doi: 10.1016/j.heliyon.2022.e12371 (PMC9800549; doi:10.1016/j.heliyon.2022.e12371)
Supplement: DN Care Survey English version 1_ 2015 [file mmc2.docx]

**Diabetic Nephropathy Care Survey**

Participant code: __________________________ Date of Survey: _____________________

## SECTION 1: DEMOGRAPHIC AND SOCIOECONOMIC CHARACTERISTICS

| Date of Birth:  / / .  (dd/mm/yyyy) | Gender:  □1 Male  □0 Female | Race:  □1 Chinese  □2 Malay  □3 Indian  □4 Others:  _________________ | Marital Status:  □1 Single  □2 Married  □3 Divorced/Separated  □4 Widowed |
| --- | --- | --- | --- |
| Highest educational level:  □1 Primary or lower  □2 Secondary or lower  □3 Post-secondary (non-tertiary) general or  vocational  □4 Polytechnic diploma, professional  qualifications, or other equivalent  qualification  □5 University degree  □6 Postgraduate degree | |  |  |
|  |  | Type of houses currently living in:  □1 HDB 1- or 2-Room  □2 HDB 3-Room  □3 HDB 4-Room  □4 HDB 5-Room / Executive  □5 Private flat / Condominium  □6 Landed Property | |
| Employment Status  □1 Working full-time  □2 Working part-time  □3 Not working (e.g. Student, retirees,  housewives & unemployed) | | Language you feel most comfortable in speaking:  □1 English  □2 My mother tongue(Please specify:_____________)  □3 Both English and my mother tongue | |
| Financial Status:  (Describe your household’s financial situation right now.)  □1 After paying the bills, you still **have enough money** for special things that you want.  □2 You have enough money to pay the bills, but **little spare money** to buy extra or special things.  □3 You have money to pay the bills, but only because you **have to cut back on things**.  □4 You are **having difficulty paying the bills**, no matter what you do. | | | |

## SECTION 2: HISTORY OF USING TCM

1. Have you ever seen a TCM physician before?

□1 Yes

□0 No **[ Skip the rest of this section ]**

1. Are you currently seeing a TCM physician?

□1 Yes

□0 No **[ Skip to question 5** **]**

1. Are you seeing your TCM physician for diabetes nephropathy or for a different condition

□1 Diabetes Nephropathy

□2 Other conditions (please specify __________________________)  **[ Skip to question 5** ]

1. What type of treatment are you receiving for your Diabetes Nephropathy?

□1 Acupuncture

□2 Herbal Medicine

□3 Moxibustion

□4 Cupping

□5 Others. (Please specify_____________________________)

1. Which type of TCM user do you consider yourself to be?

□1 A regular user for all or most of my health problems

□2 A regular user for some specific problems (please specify the problems(s)___________________)

□3 An ad hoc user

# SECTION 3: YOUR HEALTH AND WEL-BEING

**This questionnaire asks for your views about your health. This information will help keep track of how you feel and how well you are able to do your usual activities.**

**For each of the following questions, please mark an X in the one box that best describes your answer.**

1. In general, would you say your health is:

| Excellent | Very Good | Good | Fair | Poor |
| --- | --- | --- | --- | --- |
| □1 | □2 | □3 | □4 | □5 |

1. **Compared to one year ago**, how would you rate your health in general **now**?

| Much better now | Somewhat better now | About the same | Somewhat worse now | Much worse now |
| --- | --- | --- | --- | --- |
| □1 | □2 | □3 | □4 | □5 |

The following questions are about activities you might do during a typical day. Does your health NOW limit you in these activities? If so, how much?

|  | Yes. Limited  a lot | Yes. Limited  a little | No. Not limited at all |
| --- | --- | --- | --- |
| 1. **Vigorous activities**, such as running, lifting heavy objects, participating in strenuous sports | ****1 | ****2 | ****3 |
| 1. **Moderate activities**, such as moving a table, pushing a vacuum cleaner, bowling or playing golf | ****1 | ****2 | ****3 |
| 1. Lifting or carrying groceries | ****1 | ****2 | ****3 |
| 1. Climbing **several** flights of stairs | ****1 | ****2 | ****3 |
| 1. Climbing **one** flight of stairs | ****1 | ****2 | ****3 |
| 1. Bending, kneeling or stooping | ****1 | ****2 | ****3 |
| 1. Walking **more than a mile (about 1.6 kilometers)** | ****1 | ****2 | ****3 |
| 1. Walking **several blocks** | ****1 | ****2 | ****3 |
| 1. Walking **one block** | ****1 | ****2 | ****3 |
| 1. Bathing or dressing yourself | ****1 | ****2 | ****3 |

During the **past 4 weeks**, have you had any of the following problems with your work or other regular daily activities **as a result of your physical health**?

|  | Yes | No |
| --- | --- | --- |
| 1. Cut down the amount of time you spent on work or other activities | ****1 | ****2 |
| 1. **Accomplished less** than you would like | ****1 | ****2 |
| 1. Were limited in the **kind** of work or other activities | ****1 | ****2 |
| 1. Had **difficulty** performing the work or other activities (for example, it took extra effort) | ****1 | ****2 |

During the **past 4 weeks**, have you had any of the following problems with your work or other regular daily activities **as a result of any emotional problems** (such as feeling depressed or anxious?)

|  | Yes | No |
| --- | --- | --- |
| 1. Cut down the **amount of time** you spent on work or other activities | ****1 | ****2 |
| 1. **Accomplished less** than you would like | ****1 | ****2 |
| 1. Didn’t do work or other activities as **carefully** as usual | ****1 | ****2 |

1. During the **past 4 weeks**, to what extent has your physical health or emotional problems interfered with your normal social activities with family, friends, neighbors, or groups?

| Not at all | Slightly | Moderately | Quite a bit | Extremely |
| --- | --- | --- | --- | --- |
| □1 | □2 | □3 | □4 | □5 |

1. How much **bodily** pain have you had during the **past 4 weeks**?

| None | Very mild | Mild | Moderate | Severe | Very severe |
| --- | --- | --- | --- | --- | --- |
| □1 | □2 | □3 | □4 | □5 | □6 |

1. During the **past 4 weeks**, how much did **pain** interfere with your normal work (including both work outside the home and housework)?

| Not at all | A little bit | Moderately | Quite a bit | Extremely |
| --- | --- | --- | --- | --- |
| □1 | □2 | □3 | □4 | □5 |

These questions are about how you feel and how things have been with you **during the past 4 weeks**. For each question, please give the one answer that comes closest to the way you have been feeling.

How much of the time during the past 4 weeks…

|  | All of the Time | Most of the Time | A Good Bit of the Time | Some of the Time | A little of the Time | None of the Time |
| --- | --- | --- | --- | --- | --- | --- |
| 1. Did you feel full of pep (energy) | ****1 | ****2 | ****3 | ****4 | ****5 | ****6 |
| 1. Have you been a very nervous person? | ****1 | ****2 | ****3 | ****4 | ****5 | ****6 |
| 1. Have you felt so down in the dumps that nothing could cheer you up? | ****1 | ****2 | ****3 | ****4 | ****5 | ****6 |
| 1. Have you felt calm and peaceful? | ****1 | ****2 | ****3 | ****4 | ****5 | ****6 |
| 1. Did you have a lot of energy? | ****1 | ****2 | ****3 | ****4 | ****5 | ****6 |
| 1. Have you felt downhearted and blue? | ****1 | ****2 | ****3 | ****4 | ****5 | ****6 |
| 1. Did you feel worn out? | ****1 | ****2 | ****3 | ****4 | ****5 | ****6 |
| 1. Have you been a happy person? | ****1 | ****2 | ****3 | ****4 | ****5 | ****6 |
| 1. Did you feel tired? | ****1 | ****2 | ****3 | ****4 | ****5 | ****6 |

1. During the **past 4 weeks**, how much of the time has your **physical health or emotional problems** interfered with your social activities (like visiting with friends, relatives, etc.)?

| All of the  time | Most of the time | Some of the time | A little of the time | None of the time |
| --- | --- | --- | --- | --- |
| □1 | □2 | □3 | □4 | □5 |

How **TRUE or FALSE** is each of the following statements for you?

|  | Definitely True | Mostly True | Don’t Know | Mostly False | Definitely False |
| --- | --- | --- | --- | --- | --- |
| 1. I seem to get sick a little easier than other people | ****1 | ****2 | ****3 | ****4 | ****5 |
| 1. I am as healthy as anybody I know | ****1 | ****2 | ****3 | ****4 | ****5 |
| 1. I expect my health to get worse | ****1 | ****2 | ****3 | ****4 | ****5 |
| 1. My health is excellent | ****1 | ****2 | ****3 | ****4 | ****5 |

## SECTION 4: PERCEPTION OF DOCTOR-PATIENT INTERACTION

Participant Code:________________________ Date of Survey Done:_______________________

The following questions are about your **recent visits to your doctor for your diabetic nephropathy**. Please choose the answer that best reflects how your feel about the visits.

1. Overall how would you rate your experience of the interaction with your doctor?

□1 Excellent

□2 Very good

□3 Good

□4 Fair

□5 Poor

1. How did you feel about the waiting time to see your doctor during your most recent visit to your doctor?

□1 Longer than I expected

□2 Just right

□3 Shorter than I expected

1. How did you feel about the consultation time of this visit?

□1 Longer than I expected

□2 Just right

□3 Shorter than I expected

***Below is a list of statements some patients have used to describe the interaction with their doctors. Please indicate your agreement with each of them for your most recent interaction with your doctor with 1=strongly disagree and 5 being strongly agree. There is no right or wrong answer. Please CIRCLE the number that best reflects your own experience and feeling. The information you provide will be kept strictly confidential.***

|  | Strongly disagree | Disagree | Neutral | Agree | Strongly agree | Not Applicable |
| --- | --- | --- | --- | --- | --- | --- |
| 4. The doctor told me the name of my illness in words that I could understand | **1** | **2** | **3** | **4** | **5** | **6** |
| 5. After talking with the doctor, I know just how serious my illness is | **1** | **2** | **3** | **4** | **5** | **6** |
| 6. After talking with the doctor, I have a good idea of what changes to expect in my health over the next few weeks and months | **1** | **2** | **3** | **4** | **5** | **6** |
| 7. The doctor told me all I wanted to know about my illness | **1** | **2** | **3** | **4** | **5** | **6** |
| 8. The doctor is very good at explaining the reasons for medical tests | **1** | **2** | **3** | **4** | **5** | **6** |
|  | Strongly disagree | Disagree | Neutral | Agree | Strongly agree | Not Applicable |
| 9. The doctor told me how being sick will affect my ability to do work or my daily life | **1** | **2** | **3** | **4** | **5** | **6** |
| 10. The doctor has relieved my worries about being seriously ill | **1** | **2** | **3** | **4** | **5** | **6** |
| 11. The doctor told me what the medicine he prescribed would do for me | **1** | **2** | **3** | **4** | **5** | **6** |
| 12. I feel I understand pretty well the doctor’s plan for helping me | **1** | **2** | **3** | **4** | **5** | **6** |
| 13. The doctor gave me a chance to say what was really on my mind | **1** | **2** | **3** | **4** | **5** | **6** |
| 14. I really felt understood by my doctor | **1** | **2** | **3** | **4** | **5** | **6** |
| 15. After talking to the doctor, I felt much better about my problems | **1** | **2** | **3** | **4** | **5** | **6** |
| 16. I felt that this doctor really knew how upset I was about my pain | **1** | **2** | **3** | **4** | **5** | **6** |
| 17. I felt free to talk to my doctor about my private thoughts | **1** | **2** | **3** | **4** | **5** | **6** |
| 18. I felt this doctor accepted me as a person | **1** | **2** | **3** | **4** | **5** | **6** |
| 19. I felt that this doctor did not take my problems very seriously | **1** | **2** | **3** | **4** | **5** | **6** |
| 20. This doctor was not friendly to me | **1** | **2** | **3** | **4** | **5** | **6** |
| 21. The doctor I saw today would be someone I would trust with my life | **1** | **2** | **3** | **4** | **5** | **6** |
| 22. The doctor gave me a thorough checkup | **1** | **2** | **3** | **4** | **5** | **6** |
| 23. The doctor was too rough when he examined me | **1** | **2** | **3** | **4** | **5** | **6** |
| 24. The doctor looked into all the problems I mentioned | **1** | **2** | **3** | **4** | **5** | **6** |
| 25. I was satisfied with the doctor’s decision about what medicines I needed to take | **1** | **2** | **3** | **4** | **5** | **6** |
| 26. I feel the doctor did not spend enough time with me | **1** | **2** | **3** | **4** | **5** | **6** |
| 27. The doctor seemed rushed during his examination of me | **1** | **2** | **3** | **4** | **5** | **6** |
| 28. The doctor gave directions too fast when he examined me | **1** | **2** | **3** | **4** | **5** | **6** |
| 29. The doctor seemed to know what he was doing during the examination | **1** | **2** | **3** | **4** | **5** | **6** |

30. How long have you seen this doctor for your diabetic condition?

□1 Less than a year

□2 More than 1 year but less than 2 years

□3 More than 2 years but less than 5 years

□4 More than 5 years

## SECTION 5: PERCEPTION OF DOCTOR-PATIENT INTERACTION - TCM VISIT

Participant Code:________________________

Date of TCM Visit:_______________________ Date of Survey Done:_______________________

The following questions are about your **recent visits to your doctor for your diabetic nephropathy**. Please choose the answer that best reflects how your feel about the visits.

1. Overall how would you rate your experience of the interaction with your doctor?

□1 Excellent

□2 Very good

□3 Good

□4 Fair

□5 Poor

1. How did you feel about the waiting time to see your doctor during your most recent visit to your doctor?

□1 Longer than I expected

□2 Just right

□3 Shorter than I expected

1. How did you feel about the consultation time of this visit?

□1 Longer than I expected

□2 Just right

□3 Shorter than I expected

***Below is a list of statements some patients have used to describe the interaction with their doctors. Please indicate your agreement with each of them for your most recent interaction with your doctor with 1=strongly disagree and 5 being strongly agree. There is no right or wrong answer. Please CIRCLE the number that best reflects your own experience and feeling. The information you provide will be kept strictly confidential.***

|  | Strongly disagree | Disagree | Neutral | Agree | Strongly agree | Not Applicable |
| --- | --- | --- | --- | --- | --- | --- |
| 4. The doctor told me the name of my illness in words that I could understand | **1** | **2** | **3** | **4** | **5** | **6** |
| 5. After talking with the doctor, I know just how serious my illness is | **1** | **2** | **3** | **4** | **5** | **6** |
| 6. After talking with the doctor, I have a good idea of what changes to expect in my health over the next few weeks and months | **1** | **2** | **3** | **4** | **5** | **6** |
| 7. The doctor told me all I wanted to know about my illness | **1** | **2** | **3** | **4** | **5** | **6** |
|  | Strongly disagree | Disagree | Neutral | Agree | Strongly agree | Not Applicable |
| 8. The doctor is very good at explaining the reasons for medical tests | **1** | **2** | **3** | **4** | **5** | **6** |
| 9. The doctor told me how being sick will affect my ability to do work or my daily life | **1** | **2** | **3** | **4** | **5** | **6** |
| 10. The doctor has relieved my worries about being seriously ill | **1** | **2** | **3** | **4** | **5** | **6** |
| 11. The doctor told me what the medicine he prescribed would do for me | **1** | **2** | **3** | **4** | **5** | **6** |
| 12. I feel I understand pretty well the doctor’s plan for helping me | **1** | **2** | **3** | **4** | **5** | **6** |
| 13. The doctor gave me a chance to say what was really on my mind | **1** | **2** | **3** | **4** | **5** | **6** |
| 14. I really felt understood by my doctor | **1** | **2** | **3** | **4** | **5** | **6** |
| 15. After talking to the doctor, I felt much better about my problems | **1** | **2** | **3** | **4** | **5** | **6** |
| 16. I felt that this doctor really knew how upset I was about my pain | **1** | **2** | **3** | **4** | **5** | **6** |
| 17. I felt free to talk to my doctor about my private thoughts | **1** | **2** | **3** | **4** | **5** | **6** |
| 18. I felt this doctor accepted me as a person | **1** | **2** | **3** | **4** | **5** | **6** |
| 19. I felt that this doctor did not take my problems very seriously | **1** | **2** | **3** | **4** | **5** | **6** |
| 20. This doctor was not friendly to me | **1** | **2** | **3** | **4** | **5** | **6** |
| 21. The doctor I saw today would be someone I would trust with my life | **1** | **2** | **3** | **4** | **5** | **6** |
| 22. The doctor gave me a thorough checkup | **1** | **2** | **3** | **4** | **5** | **6** |
| 23. The doctor was too rough when he examined me | **1** | **2** | **3** | **4** | **5** | **6** |
| 24. The doctor looked into all the problems I mentioned | **1** | **2** | **3** | **4** | **5** | **6** |
| 25. I was satisfied with the doctor’s decision about what medicines I needed to take | **1** | **2** | **3** | **4** | **5** | **6** |
| 26. I feel the doctor did not spend enough time with me | **1** | **2** | **3** | **4** | **5** | **6** |
| 27. The doctor seemed rushed during his examination of me | **1** | **2** | **3** | **4** | **5** | **6** |
| 28. The doctor gave directions too fast when he examined me | **1** | **2** | **3** | **4** | **5** | **6** |
| 29. The doctor seemed to know what he was doing during the examination | **1** | **2** | **3** | **4** | **5** | **6** |

30. How long have you seen this doctor for your diabetic condition?

□1 Less than a year

□2 More than 1 year but less than 2 years

□3 More than 2 years but less than 5 years

□4 More than 5 years

***We have reached the end of the survey. Thank you very much for your time and contribution!***
