# Supplementary material for: Satisfaction of patients with diabetic kidney disease with traditional chinese medicine physician visits
Source: Heliyon. 2022 Dec 16;8(12):e12371. doi: 10.1016/j.heliyon.2022.e12371 (PMC9800549; doi:10.1016/j.heliyon.2022.e12371)
Supplement: MISS questionaires [file mmc3.docx]

| **MISS-26 Question** | | | | | |
| --- | --- | --- | --- | --- | --- |
|  | Strongly disagree | Disagree | Neutral | Agree | Strongly agree |
| **Cognitive** | | | | | |
| 1. The doctor told me the name of my illness in words that I could understand | **1** | **2** | **3** | **4** | **5** |
| 2. After talking with the doctor, I know just how serious my illness is | **1** | **2** | **3** | **4** | **5** |
| 3. After talking with the doctor, I have a good idea of what changes to expect in my health over the next few weeks and months | **1** | **2** | **3** | **4** | **5** |
| 4. The doctor told me all I wanted to know about my illness | **1** | **2** | **3** | **4** | **5** |
| 5. The doctor is very good at explaining the reasons for medical tests | **1** | **2** | **3** | **4** | **5** |
| 6. The doctor told me how being sick will affect my ability to do work or my daily life | **1** | **2** | **3** | **4** | **5** |
| 7. The doctor has relieved my worries about being seriously ill | **1** | **2** | **3** | **4** | **5** |
| 8. The doctor told me what the medicine he prescribed would do for me | **1** | **2** | **3** | **4** | **5** |
| 9. I feel I understand pretty well the doctor’s plan for helping me | **1** | **2** | **3** | **4** | **5** |
| **Affective** | | | | | |
| 10. The doctor gave me a chance to say what was really on my mind | **1** | **2** | **3** | **4** | **5** |
| 11. I really felt understood by my doctor | **1** | **2** | **3** | **4** | **5** |
| 12. After talking to the doctor, I felt much better about my problems | **1** | **2** | **3** | **4** | **5** |
| 13. I felt that this doctor really knew how upset I was about my pain | **1** | **2** | **3** | **4** | **5** |
| 14. I felt free to talk to my doctor about my private thoughts | **1** | **2** | **3** | **4** | **5** |
| 15. I felt this doctor accepted me as a person | **1** | **2** | **3** | **4** | **5** |
| 16. I felt that this doctor did not take my problems very seriously | **1** | **2** | **3** | **4** | **5** |
| 17. This doctor was not friendly to me | **1** | **2** | **3** | **4** | **5** |
| 18. The doctor I saw today would be someone I would trust with my life | **1** | **2** | **3** | **4** | **5** |
| **Behavioural** | | | | | |
| 19. The doctor gave me a thorough checkup | **1** | **2** | **3** | **4** | **5** |
| 20. The doctor was too rough when he examined me | **1** | **2** | **3** | **4** | **5** |
| 21. The doctor looked into all the problems I mentioned | **1** | **2** | **3** | **4** | **5** |
| 22. I was satisfied with the doctor’s decision about what medicines I needed to take | **1** | **2** | **3** | **4** | **5** |
| 23. I feel the doctor did not spend enough time with me | **1** | **2** | **3** | **4** | **5** |
| 24. The doctor seemed rushed during his examination of me | **1** | **2** | **3** | **4** | **5** |
| 25. The doctor gave directions too fast when he examined me | **1** | **2** | **3** | **4** | **5** |
| 26. The doctor seemed to know what he was doing during the examination | **1** | **2** | **3** | **4** | **5** |
